# Association of Headache Disorders and the Risk of Dementia: Meta-Analysis of Cohort Studies

**DOI:** 10.3389/fnagi.2022.804341

**Published:** 2022-02-11

**Authors:** Huiling Qu, Shida Yang, Zhicheng Yao, Xiaoyu Sun, Huisheng Chen

**Affiliations:** ^1^Department of Neurology, The General Hospital of Northern Theater Command, Shenyang, China; ^2^Department of Laboratory Medicine, The People's Hospital of Liaoning Province, Shenyang, China; ^3^Department of Neurology, The People's Hospital of Liaoning Province, Shenyang, China

**Keywords:** headache, dementia, Alzheimer's disease, vascular dementia, meta-analysis

## Abstract

**Objectives:**

The purpose of this meta-analysis is to assess whether there is an association between headache disorders and all-cause dementia, Alzheimer's disease (AD), and vascular dementia (VaD).

**Methods:**

PubMed, Cochrane Library, Embase, and Web of Science were searched for cohort studies published from database inception to October 8, 2021, using medical subject headings (MeSH) and keywords. All statistical analyses were performed using Stata statistical software version 14.0. If *P* > 0.1 and *I*^2^ ≤ 50%, a fixed-effects model was adopted. If *I*^2^ > 50% (which indicated great heterogeneity), a random-effects model was adopted. The funnel plot and Egger's test were used to evaluate publication bias.

**Results:**

This meta-analysis included 12 cohort studies covering 465,358 individuals, which were published between 2001 and 2020. The pooling analysis shows that a history of any headache disorder is associated with an increased risk of all-cause dementia (OR = 1.35; 95% CI: 1.21–1.50; *I*^2^ = 81.6%, *P* < 0.001). The history of any headache was associated with an increased risk of AD (OR = 1.49; 95% CI: 1.08–2.05; *I*^2^ = 70.0%, *P* = 0.003) and VaD (OR = 1.72; 95% CI: 1.32–2.25; *I*^2^ = 0%, *P* < 0.001). In the subgroup analysis, females with a history of headache have a slightly higher risk of dementia than males (OR = 1.32; 95% CI: 1.16–1.51; *I*^2^ = 88.3%, *P* < 0.001) and the risk of dementia in the retrospective cohort was slightly higher than in the prospective cohort (OR = 1.38; 95% CI: 1.22–1.56; *I*^2^ = 83.4%, *P* < 0.001).

**Conclusions:**

Our meta-analysis shows that any headache disorder increases the risk of all-cause dementia, AD, or VaD. These findings provide evidence that headache should be recognized as an independent risk factor for dementia, AD, or VaD.

## Background

Dementia is a neurological disorder characterized by cognitive, behavioral, social, and emotional deterioration. It is a major public health problem in the world and has a high incidence rate (Van Der Steen et al., 2018). Although significant progress has been made in molecular neuroimaging, clinical pathology, and the development of biomarkers of dementia in the last decade, the results are slightly disappointing. Clinicians are still waiting for disease modification therapy of dementia (Gale et al., 2018). Therefore, if we can identify the risk factors associated with dementia early, the development of dementia might be prevented. A previous study has explored the risk factors of dementia, including age, gender, family history, rural residents, low educational level, marital status, smoking, hypertension, hyperlipidemia, diabetes, heart disease, and cerebrovascular disease (Jia et al., 2020). The impact of headache on dementia has not been noted.

Headaches (including migraine, tension headache, and drug overuse headache) are associated with a high incidence rate, low quality of life, low productivity, and high economic costs. A global disease burden study listed headache as the second largest cause of disability in the world. Among people aged 15–49, the incidence of migraine is the third highest (Saylor and Steiner, 2018). Migraine can be regarded as a high signal of white matter-related risk factors (Kruit et al., 2004). Patients with headache are more likely to have extensive white matter hyperintensity (WMH) than patients without headache (Honningsvåg et al., 2018). White matter hyperintensity may be associated with dementia. Therefore, we speculate that headache may be associated with an increased risk of dementia, and we systematically reviewed the existing population-based longitudinal evidence to determine the association between headache disorder and the risk of all-cause dementia, Alzheimer's disease (AD), or vascular dementia (VaD).

## Methods

This meta-analysis was conducted in accordance with the guidelines of the Preferred Reporting Items for Systematic Reviews and Meta-Analyses (PRISMA) (Page et al., 2021). The protocol was pre-registered in the International Prospective Register of Systematic Reviews (PROSPERO) platform, and the approval number is CRD42021283921.

### Data Sources and Searches

PubMed, Cochrane Library, Embase, and Web of Science were searched for cohort studies published from database inception to October 8, 2021. There were no language restrictions, and the search strategy combined the use of medical subject headings (MeSH) and keywords. The search terms included dementia, AD, VaD, headache, head pain, migraine, and cohort studies. The full search strategy of PubMed is included in Supplementary Table 1. The reference lists of included cohort studies and other published meta-analyses were also examined to identify relevant trials.

### Eligibility Criteria

The trials were included on the basis of the following eligibility criteria: (1) cohort studies or nested case-control studies based on cohort trials; (2) investigations of the association of headache disorders with the risk of incident all-cause dementia, AD, or VaD. In this meta-analysis, “any headache” was defined as “patients who suffered from any type of primary headache in the past.” All-cause dementia was chosen as the primary outcome, AD, and VaD as the secondary outcomes.

Trials were excluded if they did not provide an odds ratio (OR) estimate with corresponding 95% confidence interval (CI). If more than one study reported data from the same cohort, we included the study with the longest follow-up or the largest number of participants. Furthermore, the following articles were also excluded: conference abstracts, study protocols, duplicate publications, and studies with no outcomes of interest.

### Study Selection

Study selection was performed by two reviewers (HLQ and ZCY) who independently screened the literature based on the eligibility and exclusion criteria. Duplicate and irrelevant articles were first excluded according to their titles and abstracts. Thereafter, the full texts of the potentially eligible articles were downloaded and read to identify all eligible studies. Any disagreements were resolved by the third reviewer (SDY), who acted as an arbiter.

### Data Extraction

Data extraction was performed independently by the two above-mentioned reviewers (HLQ and ZCY) who consulted the guidelines on data extraction for systematic reviews and meta-analysis (Taylor et al., 2021). They used predesigned forms for extracting data including the first author, year of publication, study type, sample size, follow-up years, age, diagnosis of migraine/dementia, headache type, dementia type, and confounders adjusted. Disagreements were resolved by discussion with SDY to reach a consensus.

### Risk of Bias Assessment

The Newcastle-Ottawa scale (NOS) was used to assess the quality of cohort studies (Wells et al., 2014). Stars ranged from 0 to 9 points for cohort studies, four stars for selection of participants and measurement of exposure, two stars for comparability, and three stars for assessment of outcomes and adequacy of follow-up, with more stars indicating higher quality of study. Scores of 0–3, 4–6, and 7–9 were considered to indicated low, moderate, and high quality, respectively.

### Statistical Analysis

The adjusted OR and 95% CI from each trial were used to assess the association between headache disorders and risk of dementia, AD, or VaD. The χ^2^-test and the *I*^2^-values were used to evaluate heterogeneity. If *P* > 0.1 and *I*^2^ ≤ 50%, a fixed-effects model was adopted. If *I*^2^ > 50% (which indicated great heterogeneity), a random-effects model was adopted. The sensitivity analysis was performed by excluding one study each time and rerunning to verify the robustness of the overall effects. The funnel plot was visually inspected to confirm publication bias, and Egger's regression test was used to statistically assess publication bias. We conducted a subgroup analysis based on gender and research type. All statistical analyses were performed using Stata statistical software version 14.0 (Stata Corp, College Station, Texas).

## Results

### Literature Search

**Figure 1:**
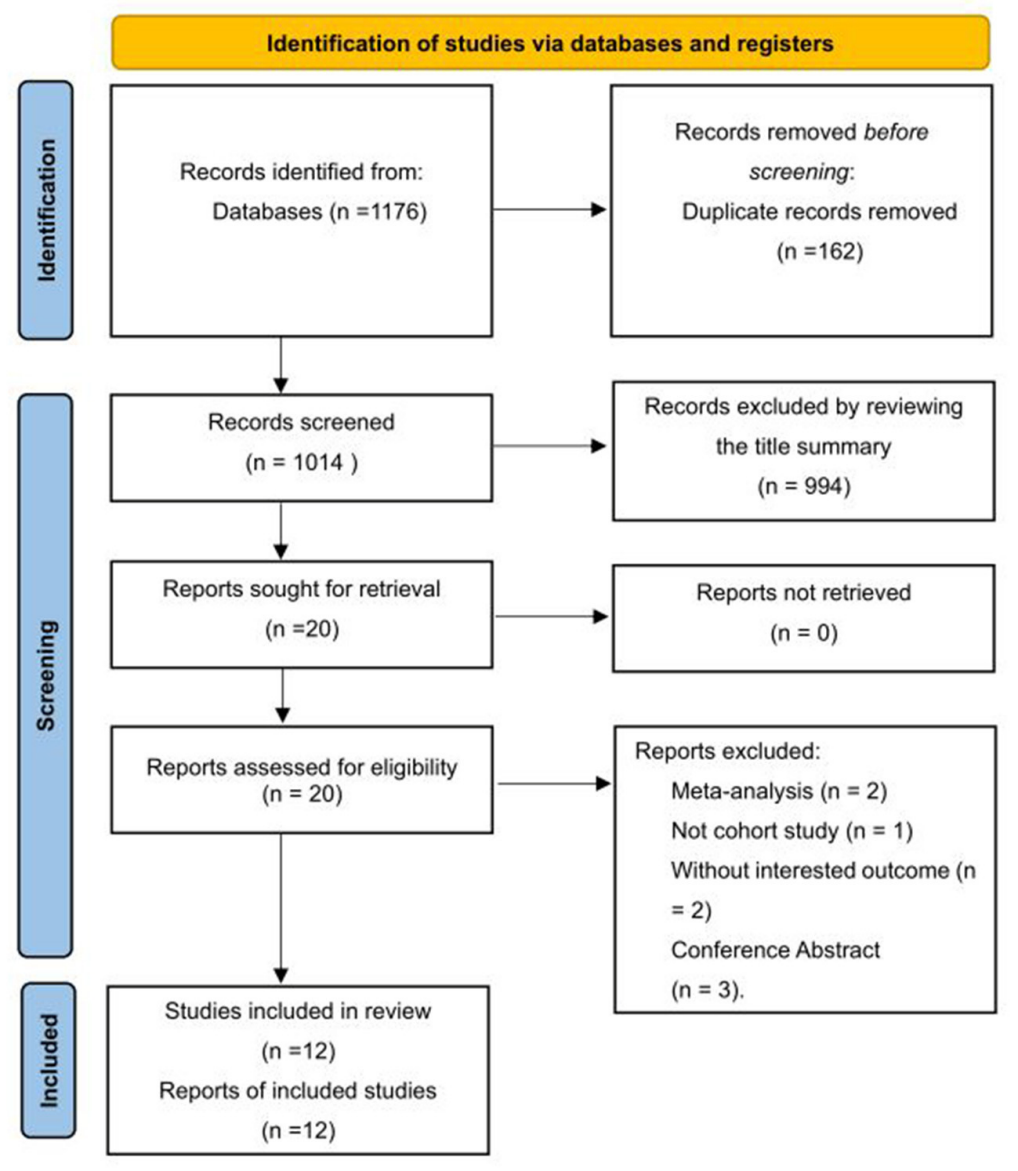
Studies screening process.

The systematic search of cohort studies published before October 8, 2021, identified 1,176 results. After title and abstract screening, 20 articles were considered potentially relevant. Twelve studies (Tyas et al., 2001; Chuang et al., 2013; Hagen et al., 2014; Røttereng et al., 2015; Yang et al., 2016; Tzeng et al., 2017; Yin et al., 2018; Kostev et al., 2019; Lee et al., 2019; Morton et al., 2019; George et al., 2020; Islamoska et al., 2020) were included after full text review, of which 11 reported the incidence of dementia or composite of AD or VaD on follow-up, and one study (Tyas et al., 2001) reported the incidence of AD only. The selection process is presented in Figure 1.

### Study Characteristics

This meta-analysis included 12 cohort studies covering 465,358 individuals, which were published between 2001 and 2020. Four studies were retrospective cohort studies, while the other eight were prospective cohort studies. All individuals in these cohorts were at least 20 years old at the beginning of follow-up, and had clear diagnostic criteria for dementia. The average follow-up time ranged from 5 to 22 years. The adjusted estimates were available for almost all studies even though the adjusted confounders are slightly different. The main characteristics of the included trials are shown in Table 1.

**Table 1:** Basic characteristics of the included studies.

**Author**	**Year**	**Country**	**Study type**	**Sample size**	**Follow-up years**	**Age (years)**	**Diagnosis of migraine/ dementia**	**Headache type**	**Dementia type**	**Confounders adjusted**	**NOS scores**
George et al. (2020)	2020	USA	Prospective cohort	Total: 11,252 Migraine: 1,397, No migraine: 9,855	21 averages	51~70	ICHD-II/ICD-9	Migraine/ Severe Non-migraine Headache	All-cause dementia	Age, sex, race, center, APOE ε4, income, education, BMI, smoking status, hypertension, diabetes, prevalent CHD, drinking status, HDL cholesterol, and total cholesterol	9
Islamoska et al. (2020))	2020	Denmark	Retrospective cohort	Total: 62,578 Migraine: 10857, No migraine: 51721	3.6~11.2	49 averages	ICD-8/ICD-10	Migraine	All-cause dementia	Sex, country of origin, marital status, educational level, headache diagnoses, psychiatric morbidities, and Charlson Comorbidity Index (CCI)	8
Morton et al. (2019)	2019	Canada	Prospective cohort	Total: 679	5 averages	≥65	ICHD-II/DSM-IV	Migraine	All-cause dementia, Alzheimer's disease (AD), Vascular dementia (VaD)	Age and education	7
Lee et al. (2019)	2019	Korea	Retrospective cohort	Total:56,309 Migraine: 45,752, No migraine: 10,557	11 averages	≥60	ICD-10	Migraine	All-cause dementia	Age, sex, income, region of residence, hypertension, diabetes, and dyslipidemia	7
Kostev et al. (2019)	2019	Germany	Retrospective cohort	Total: 7,454 Migraine: 3,727, No migraine: 3,727	10 averages	60~80	ICD-10	Migraine	All- cause dementia, Alzheimer's disease (AD), Vascular dementia (VaD)	No report	8
Yin et al. (2018)	2018	China	Retrospective cohort	Total:6,730 PHDs:1,346, No migraine: 5,384	5 averages	47.38 averages	ICD-9	Primary headache	All-cause dementia, Alzheimer's disease (AD), Vascular dementia (VaD)	Age, sex, hypertension, DM, IHD, hyperlipidemia, AF, TUD, alcoholism, obesity, PD, CVA, depression, CKD, and CAI	8
Tzeng et al. (2017)	2016	China	Retrospective cohort	Total: 14,480 Headache: 3,620 No headache: 10,860	10 averages	≥20	ICD-9	Migraines, tension-type headaches	All-cause dementia, Alzheimer's disease (AD), Vascular dementia (VaD)	Gender, age, monthly income, urbanization level, geographic region of residence, and comorbidities	7
Yang et al. (2016)	2016	China	Retrospective cohort	Total: 69,540 TTH: 13,908 No TTH: 55,632	8.14 averages	≥20	ICD-9	Tension-type headaches	All-cause dementia, Alzheimer's disease (AD), Vascular dementia (VaD)	Age, sex, diabetes, dyslipidemias, COPD, hypertension, IHD, AF, HF, stroke, depression, head injury, Parkinson's disease, and migraine	8
Røttereng et al. (2015)	2015	Norway	Retrospective cohort	Total: 16,443 Any headache: 8,676 No headache: 7,767	11 averages	≥55	ICHD-I	Any headache	All-cause dementia, Alzheimer's disease (AD), Vascular dementia (VaD)	Age, gender, level of education, comorbidity, smoking, and anxiety and depression	7
Hagen et al. (2014)	2013	Norway	Prospective cohort	Total: 51,859 Any headache: 21,871 No headache: 29,988	15 averages	≥20	ICHD-I	Any headache Migraine Non-migraine headache	All-cause dementia	Age, sex, education, total HADS score, and smoking	8
Chuang et al. (2013)	2013	China	Retrospective cohort	Total: 167,340 Migraine: 33,468 No migraine: 133,872	12 longest	42.2 means	ICD-9	Migraine	All-cause dementia	Age, sex, diabetes, hypertension, depression, head injury, and CAD	8
Tyas et al. (2001)	2001	USA	Prospective cohort	Total: 694 Migraine: 36 No migraine: 658	5 averages	74 means	NINCDS–ADRDA	Migraine	Alzheimer's disease (AD)	Age, education, and sex, occupational exposure	7

### Quality Assessment

According to NOS criteria, the average score was 7.67 of all included cohort studies, and the score for each trail was 7 or above, indicating that all cohort studies were of high quality in this meta-analysis. The scores of the included studies are shown in Table 1.

### Any Headache Disorders and Risk of All-Cause Dementia

**Figure 2:**
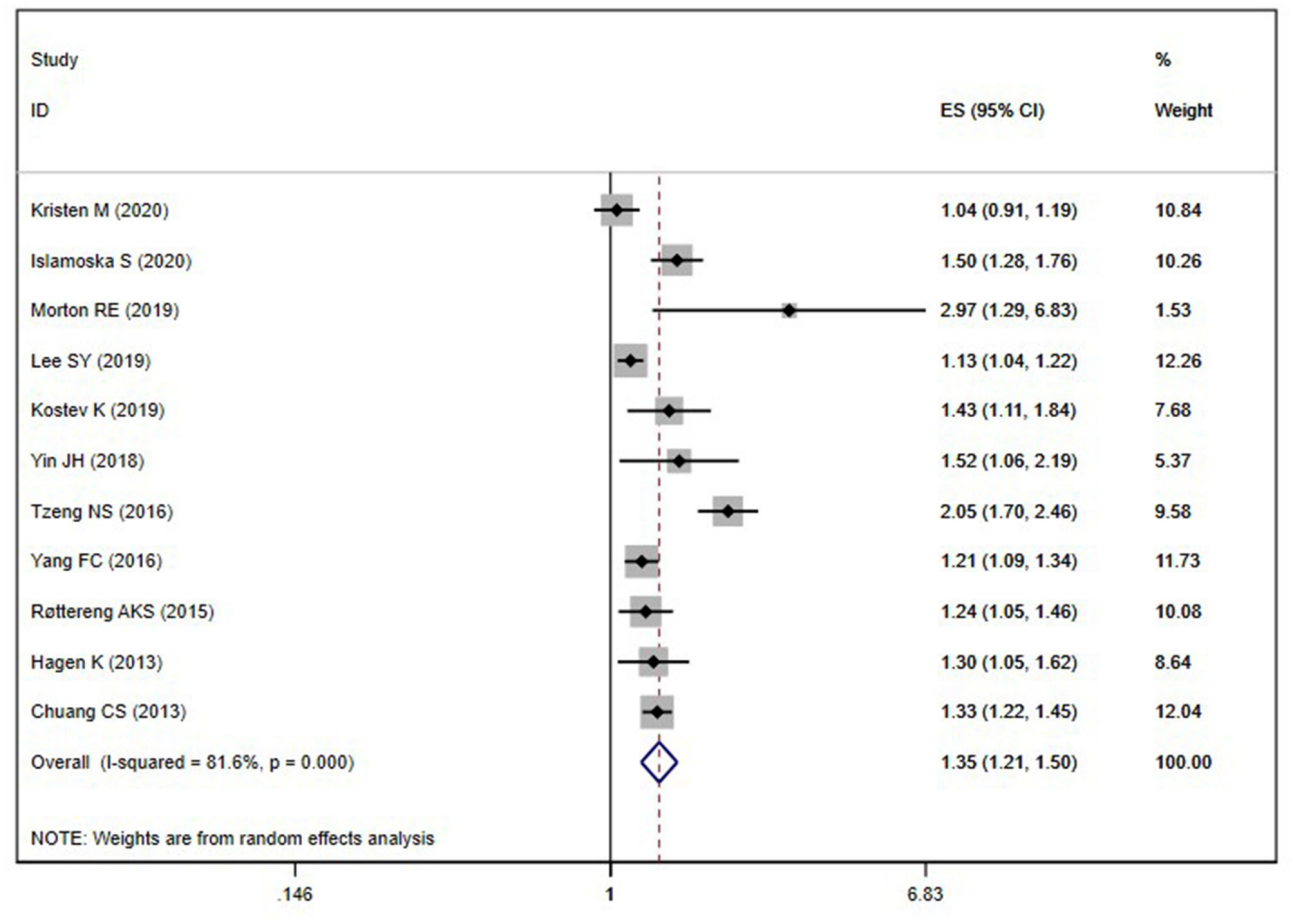
Meta-analysis of the risk of all-cause dementia caused by any headache.

Eleven cohort studies (Chuang et al., 2013; Hagen et al., 2014; Røttereng et al., 2015; Yang et al., 2016; Tzeng et al., 2017; Yin et al., 2018; Kostev et al., 2019; Lee et al., 2019; Morton et al., 2019; George et al., 2020; Islamoska et al., 2020) explored the association between a history of headache and the risk of all-cause dementia. The pooling analysis shows that a history of any headache disorder is associated with an increased risk of all-cause dementia (OR = 1.35; 95% CI: 1.21–1.50; *I*^2^ = 81.6%, *P* < 0.001; Figure 2). Sensitivity analysis showed that none of the individual studies reversed the pooled-effect size, which means that the results are robust (Supplementary Figure A).

### Any Headache Disorders and Risk of AD

**Figure 3:**
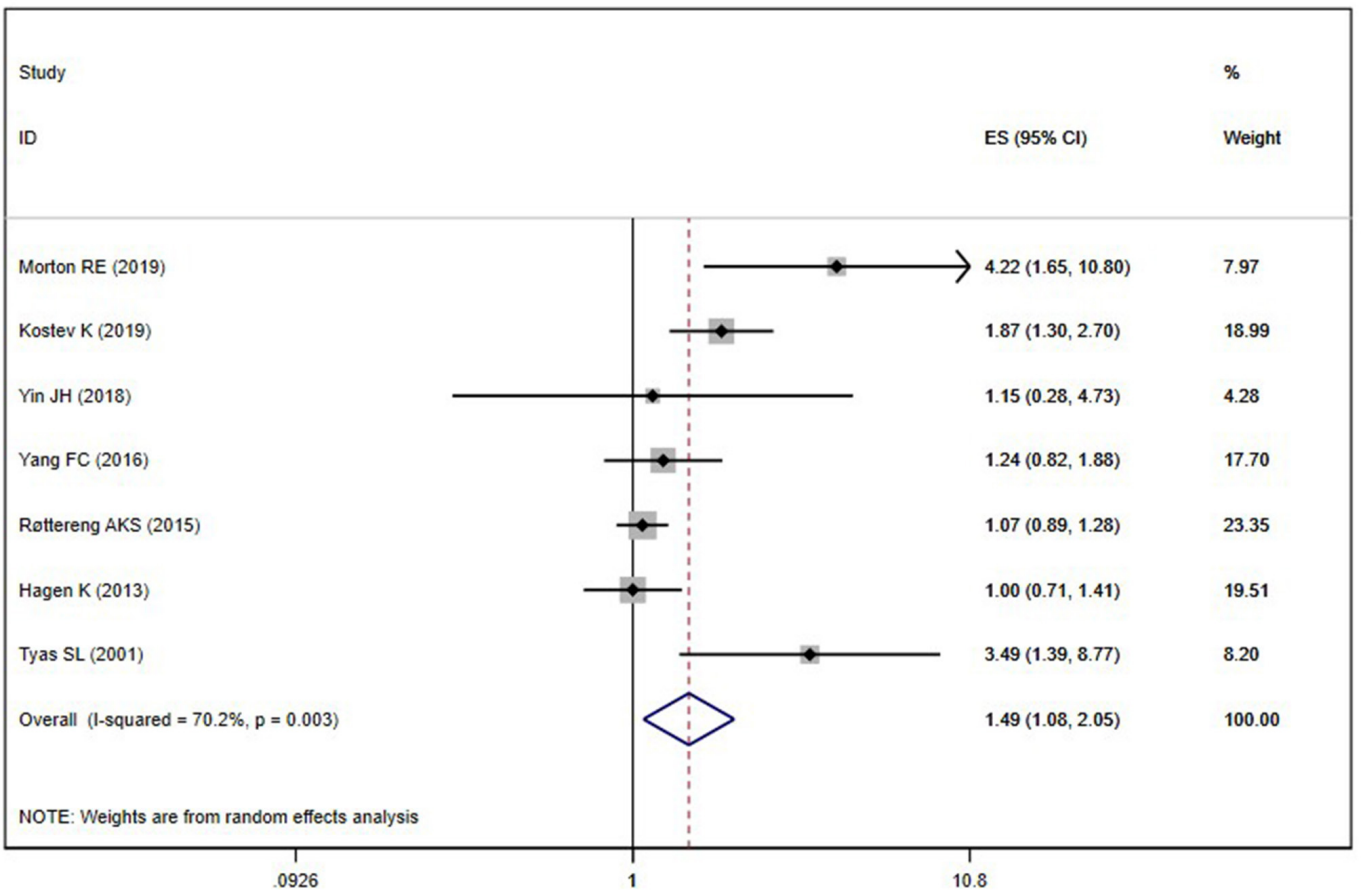
Meta-analysis of the risk of Alzheimer's disease caused by any headache.

Seven included studies (Tyas et al., 2001; Hagen et al., 2014; Røttereng et al., 2015; Yang et al., 2016; Yin et al., 2018; Kostev et al., 2019; Morton et al., 2019) assessed the association between any headache and the risk of AD. Overall, the history of any headache was associated with an increased risk of AD (OR = 1.49; 95% CI: 1.08–2.05; *I*^2^ = 70.0%, *P* = 0.003; Figure 3). Sensitivity analysis showed that none of the individual studies had reversed the pooled-effect size, which means that the results are robust (Supplementary Figure B).

### Any Headache Disorders and Risk of VaD

**Figure 4:**
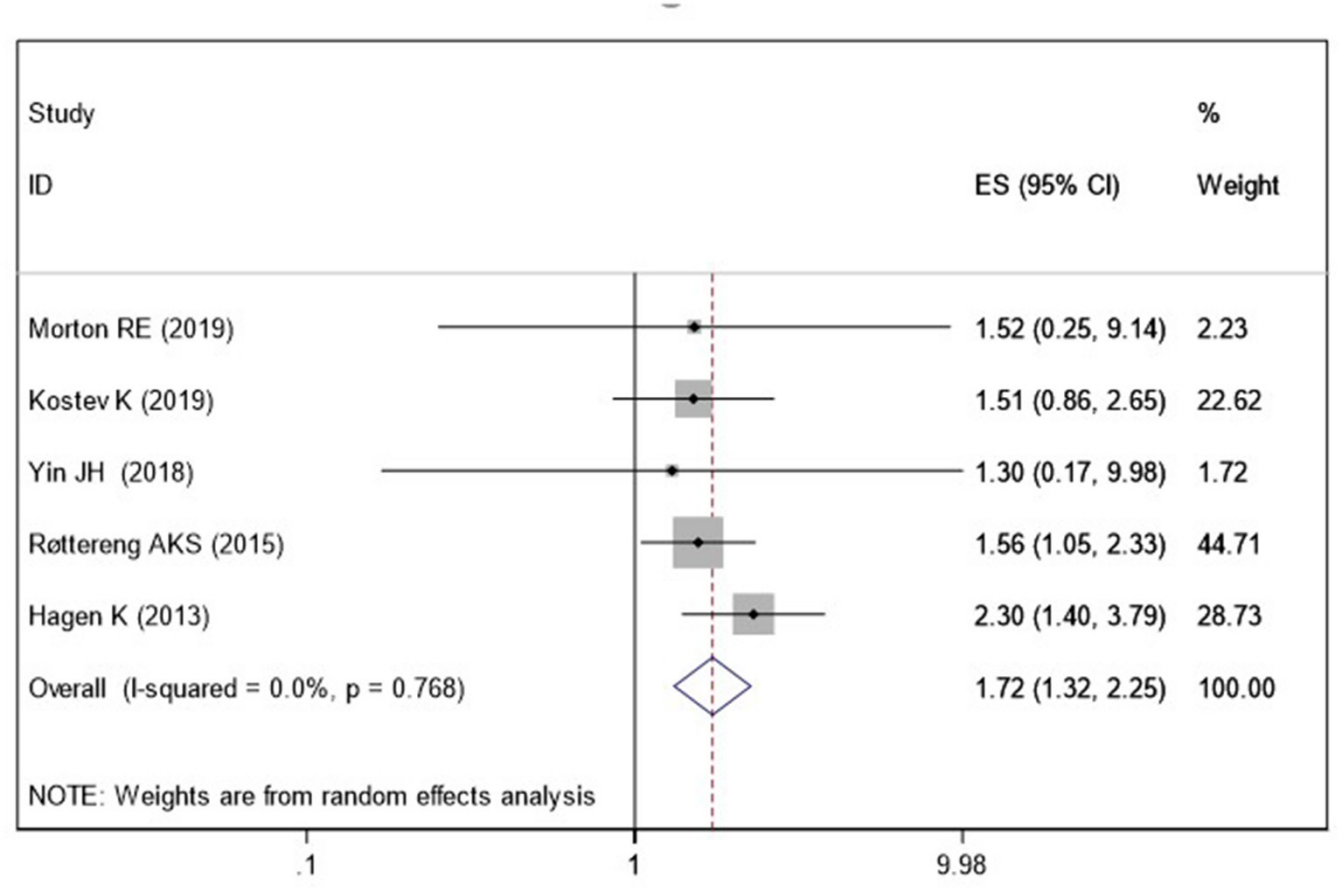
Meta-analysis of the risk of vascular dementia caused by any headache.

Five studies (Hagen et al., 2014; Røttereng et al., 2015; Yin et al., 2018; Kostev et al., 2019; Morton et al., 2019) assessed the association between a history of headache and the risk of VaD. Pooled results showed that a history of any headache disorder is associated with an increased risk of VaD (OR = 1.72; 95% CI: 1.32–2.25; *I*^2^ = 0%, *P* < 0.001; Figure 4).

### Subgroup Analysis

We conducted a subgroup analysis of gender and research type, but still did not find the origin of the high heterogeneity. In the subgroup analysis, females with a history of headaches have a slightly higher risk of dementia than males; in the prospective cohort design, there is no direct relationship between the history of headaches and the increased risk of dementia. Meanwhile, a history of migraine is associated with a higher risk of dementia (OR = 1.32; 95% CI: 1.13–1.40; *I*^2^ = 75.6%, *P* < 0.001), but the risk is lower than that of non-migraine headache patients (Table 2).

**Table 2:** Subgroup analysis for the risk of dementia in patients with headache.

**Subgroups**	**Included** **studies**	**OR]** **(95% CI)**	**Heterogeneity**	
			***I*^2^ (%)**	***P*-values**
**Sex**				
Female	7	1.32 (1.16–1.51)	88.3	0.000
Male	9	1.28 (1.09–1.50)	80.5	0.000
**Study type**				
Retrospective cohort	8	1.38 (1.22–1.56)	83.4	0.000
Prospective cohort	3	1.28 (0.94–1.76)	75.5	0.017
**Headache type**				
Migraine	7	1.26 (1.13–1.40)	75.6	0.000
Non-migraine	4	1.50(1.15–1.95)	83.1	0.003

### Publication Bias

**Figure 5:**
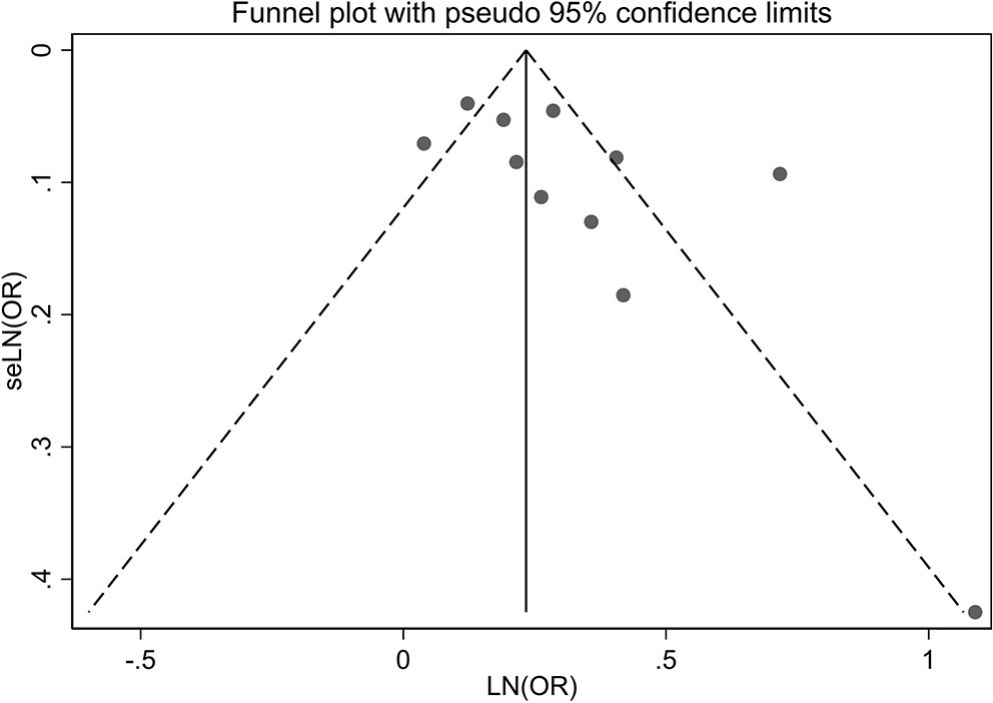
Publication bias of the risk of all-cause dementia caused by any headache.

A visual inspection of the funnel plot showed no evidence of a significant publication bias in the outcome of any headache disorders and risk of all-cause dementia (Figure 5). Egger's regression test (*P* = 0.087) likewise indicated no publication bias in our meta-analysis.

## Discussion

### Main Findings

This meta-analysis included 12 cohort studies covering 465,358 individuals, which provided a comprehensive evaluation on the association between headache and dementia. We found a significant increase in the risk of all-cause dementia, AD, or VaD among individuals with headache, with an overall 1.35-fold, 1.49-fold, or 1.72-fold increase in risk, respectively, compared with non-headache controls. This indicates that headache might be an independent risk factor for dementia.

### Interpretation of Findings

A previous review investigated the relationship between headache and dementia (Wang et al., 2018). The results showed that any headache increased the risk of all-cause dementia. However, it does not mean that headache is associated with an increased risk of all types of dementia. Moreover, it did not find a relationship between any headache and AD. In contrast, in the current analysis, we added more recent studies and analyzed the data according to the type and subgroup of dementia, so as to provide strong evidence for the association between headache and all-cause dementia, AD, or VaD. Another meta-analysis revealed that migraine is a potential risk indicator for AD and all-cause dementia (Wang et al., 2021). However, they did not find any association between migraine and the risk of VaD, which may be reasonably associated with the low number of studies included in their meta-analysis. Only five published cohort studies were identified in the review mentioned above. So, we re-analyzed the relationship between any type of headache and all-cause dementia, AD, or VaD. Our study found that any type of headache increases the risk of all-cause dementia, AD, or VaD, and the previous meta-analysis did not show these meaningful conclusions.

So far, there are few studies on the pathophysiological mechanism of the association between headache and dementia. Several brain structures involved in the pain network, such as the thalamus, insula, anterior cingulate gyrus, amygdala, and temporal cortex, also play an important role in the memory network (Apkarian et al., 2005; Svoboda et al., 2006). The overlap of the pain and memory network regions explains the potential correlation between chronic pain and memory impairment in patients with headache. The changes of hippocampal function and structure may play an important role in the pathophysiology of migraine (Maleki et al., 2013). The hippocampus is involved in memory consolidation, spatial navigation, and the stress response. Migraine is a paroxysmal disease. Each attack is accompanied by or causes many physiological and emotional stressors. Therefore, migraine attack can be regarded as a repeated stressor, causing changes in hippocampal structure and function. Some research showed that WMH is associated with an increased risk of all-cause dementia and AD (Godin et al., 2008; Bos et al., 2018; Garnier-Crussard et al., 2020). However, only a few studies have investigated the changes of the white matter microstructure and structural connectivity in migraine patients. The pathophysiology of migraine-related WMH is still poorly understood. In the current study, chronic headache may change the network of white matter by changing the mode and number of connections, resulting in the destruction of network topology. Therefore, the brain structural network of migraine patients shows abnormal overall integration between different migraine-related brain circuits adapted to long-term pain (Liu et al., 2013). Therefore, the white matter fibers of migraine patients will have abnormal changes, which may be related to the occurrence of dementia. Migraine is an independent risk factor for ischemic stroke, which may be related to the pathogenesis of VaD (Paemeleire, 2009). Migraine patients are more likely to develop psychiatric diseases, such as depression (Breslau et al., 1994; Chen et al., 2012; Rammohan et al., 2019). In particular, early depression (or depressive symptoms) has been associated with a more than two-fold increase in the risk of dementia (Katon et al., 2015; Lin et al., 2017). The possible biological mechanisms linking depression with dementia include vascular disease, changes in glucocorticoid levels, hippocampal atrophy, increased amyloid β plaque deposition, inflammatory changes, and nerve growth factor deficiency (Byers and Yaffe, 2011). In other words, patients with depression have a higher risk of dementia (Rapp et al., 2006; Wint, 2011). The number of neuritic plaques and neurofibrillary tangles in the hippocampus of AD patients with major depressive disorder confirmed by neuropathology is higher than that of AD patients who have never had a major depressive disorder in their life (Ringman et al., 2008).

In the subgroup analysis, females with a history of headaches have a slightly higher risk of dementia than males. Female are significantly more susceptible to migraine. The role of sex hormone fluctuations in promoting migraine attacks is well-known. This may be related to female sex hormones and their physiological fluctuations, which may play a role in women's susceptibility to pain hypersensitivity (Gazerani et al., 2005). In most clinical studies, migraine patients show impaired cognitive function during the interictal period (Cady and Farmer, 2013). An apolipoprotein E (ApoE) genotype is equally common in men and women, but plays a stronger role in women (Rocca et al., 2014). Apolipoprotein E is positively correlated with headache (Miao et al., 2015), which may explain why females with a history of headaches have a slightly higher risk of dementia than males.

### Implications and Limitations

Our meta-analysis summarizes the existing evidence of the association between a history of headache and the risk of all-cause dementia and shows that any headache is a risk factor for all-cause dementia. It suggests that we need to pay more attention to the dementia risk of headache patients, which is also conducive to the early identification of high-risk groups of dementia. Meanwhile, this study also has certain limitations. We only included cohort studies. This controls many confounders and hence the conclusion is reliable. Future studies can consider including case-control studies and cross-sectional studies to enrich the research types. Moreover, we did not include covariate analysis in this meta-analysis. However, the included cohort studies have controlled the adjusted confounders and thus have a well-controlled confounding bias, making the conclusions of this study reliable and facilitating translation to the clinic.

## Conclusions

This meta-analysis suggests that any headache disorder increases the risk of all-cause dementia, AD, or VaD. However, more studies are still needed to confirm the pathophysiological mechanisms underlying this phenomenon. The results of our meta-analysis can be very useful in the development of new dementia prevention and treatment strategies.

## Data Availability Statement

The datasets presented in this study can be found in online repositories. The names of the repository/repositories and accession number(s) can be found in the article/Supplementary Material.

## Author Contributions

XS and HC conceived the study. HQ and ZY collected the data and drafted the manuscript. SY revised the manuscript and language. HQ conducted the subgroup analysis. All authors have read and approved the manuscript.

## Funding

This study was supported by the Doctoral Research Start-Up Fund Project of Liaoning (No. 2019-BS-234) and the Young and Middle-Aged Scientific and Technological Innovation Talents Support Program of Shenyang (No. RC210374).

## Conflict of Interest

The authors declare that the research was conducted in the absence of any commercial or financial relationships that could be construed as a potential conflict of interest.

## Publisher's Note

All claims expressed in this article are solely those of the authors and do not necessarily represent those of their affiliated organizations, or those of the publisher, the editors and the reviewers. Any product that may be evaluated in this article, or claim that may be made by its manufacturer, is not guaranteed or endorsed by the publisher.
