# Supplementary material for: Association of Headache Disorders and the Risk of Dementia: Meta-Analysis of Cohort Studies
Source: Front Aging Neurosci. 2022 Feb 11;14:804341. doi: 10.3389/fnagi.2022.804341 (PMC8873983; doi:10.3389/fnagi.2022.804341)
Supplement: Supplementary file 1 [file Data_Sheet_1.docx]

Table 1 The full search strategy of PubMed

| No. | Query | Results |
| --- | --- | --- |
| 1 | "Dementia"[Mesh] | 181,008 |
| 2 | "Alzheimer Disease"[Mesh] | 103,079 |
| 3 | ((Dementia*[Title/Abstract])) OR (Alzheimer Dementia*[Title/Abstract]) | 124,282 |
| 4 | (("Dementia"[Mesh]) OR ("Alzheimer Disease"[Mesh])) OR (((Dementia*[Title/Abstract])) OR (Alzheimer Dementia*[Title/Abstract])) | 228,502 |
| 5 | ("Headache"[Mesh]) OR "Migraine Disorders"[Mesh] | 54,892 |
| 6 | ((Migraine*[Title/Abstract]) OR (Head pain*[Title/Abstract])) OR (Headache [Title/Abstract]) | 100,864 |
| 7 | (("Headache"[Mesh]) OR "Migraine Disorders"[Mesh]) OR (((Migraine*[Title/Abstract]) OR (Head pain*[Title/Abstract])) OR (Headache [Title/Abstract])) | 113,871 |
| 8 | "Cohort Studies"[Mesh] | 2,220,841 |
| 9 | (Cohort [Title/Abstract]) OR ("Cohort Studies"[Mesh]) | 2,486,553 |
| 10 | (((("Dementia"[Mesh]) OR ("Alzheimer Disease"[Mesh])) OR (((Dementia*[Title/Abstract])) OR (Alzheimer Dementia*[Title/Abstract]))) AND ((("Headache"[Mesh]) OR "Migraine Disorders"[Mesh]) OR (((Migraine*[Title/Abstract]) OR (Head pain*[Title/Abstract])) OR (Headache [Title/Abstract])))) AND ((Cohort [Title/Abstract]) OR ("Cohort Studies"[Mesh])) | 181 |

Figure A: Sensitivity analysis of the risk of all-cause dementia caused by any headache

Figure B Sensitivity analysis of the risk of Alzheimer's disease caused by any headache
